# The genetic relationship between extirpated and contemporary Atlantic salmon *Salmo salar* L. lines from the southern Baltic Sea

**DOI:** 10.1186/s12711-016-0208-y

**Published:** 2016-04-01

**Authors:** Rafał Bernaś, Anita Poćwierz-Kotus, Piotr Dębowski, Roman Wenne

**Affiliations:** Department of Migratory Fishes, Inland Fisheries Institute, 83-330 Rutki, Żukowo, Poland; Institute of Oceanology, Polish Academy of Sciences, 81-712 Sopot, Poland

## Abstract

**Background:**

The genetic relationship between original Atlantic salmon populations that are now extinct in the southern Baltic Sea and the present-day populations has long been controversial. To investigate and clarify this issue, we successfully genotyped individuals of the historical populations from the Oder and Vistula Rivers using DNA extracted from dried scales with the Atlantic salmon single nucleotide polymorphism array.

**Results:**

Our results showed a global *F*_ST_ of 0.2515 for all pairs of loci, which indicates a high level of genetic differentiation among the groups analyzed in this study. Pairwise *F*_ST_ values were significant for all comparisons and the highest values were found between present-day reintroduced Slupia River salmon and extinct Vistula River Atlantic salmon. Bayesian analysis of genetic structure revealed the existence of substructures in the extirpated Polish populations and three main clades among studied stocks.

**Conclusions:**

The historical salmon population from the Oder River was genetically closer to present-day salmon from the Neman River than to the historical salmon from the Vistula River. Vistula salmon clearly separated from all other analyzed salmon stocks. It is likely that the origins of the Atlantic salmon population from the Morrum River and the Polish historical native populations are different.

**Electronic supplementary material:**

The online version of this article (doi:10.1186/s12711-016-0208-y) contains supplementary material, which is available to authorized users.

## Background

Currently, the European distribution of the Atlantic salmon *Salmo salar* L. ranges from northern Portugal to the Pechora River in northwest Russia and encompasses Iceland, the British Isles and the Baltic Sea [1]. Baltic salmon populations reproduce in about 45 river systems of which at least 29 hold native salmon populations or partly mixed following stocking practices [2]. The native Atlantic salmon in Poland became extinct in the middle of the twentieth century. Salmon disappeared from the upper Vistula River in the 1950s, from its lower course after the Włocławek Dam was opened in the 1960s and from most Pomeranian rivers during the same period. Salmon also disappeared from the Drawa River (Oder basin) at the end of the 1980s.

Historically, the largest population of salmon ascended the Vistula River and migrated to the tributaries of the upper Vistula River [3]. In the Oder River, salmon migrated upstream to the mountain tributaries, but due to dam construction on these rivers during the second half of the twentieth century, salmon spawning grounds were observed mainly in the Drawa River [3]. The present Polish wild salmon population in the Slupia River originated from the Latvian Daugava River, from which individuals were collected for a recovery program that was launched in the 1990s [3]. To date, the genetic make-up of the Polish salmon breeding population is still very similar to that of the Daugava stock [4].

Ongoing discussions about the post-glacial colonization routes that were followed by the Baltic salmon (*Salmo salar*) have, to date, resulted in three hypotheses. The first one argues that Baltic salmon may have derived from a refugial population that survived in eastern pre-glacial lakes and colonized the Baltic basin before a marine strait connected the Baltic Sea to the North Sea [5, 6]. The second hypothesis suggests that Baltic salmon have a western Atlantic origin [7], whereas the third one proposes that colonization spread from both east and west areas [8]. Knowledge on how the southern Baltic Sea was colonized by Atlantic salmon populations is still scarce and the only published results concern populations from the southern Swedish rivers, Morrum and Eman [9] and the southeast Neman River [10]. Until now, extinction of Polish populations has prevented analysis of these lines. However, preserved salmon scales and tissue fragments that were collected before 1970 can be used to extract DNA samples.

**Fig. 1:**
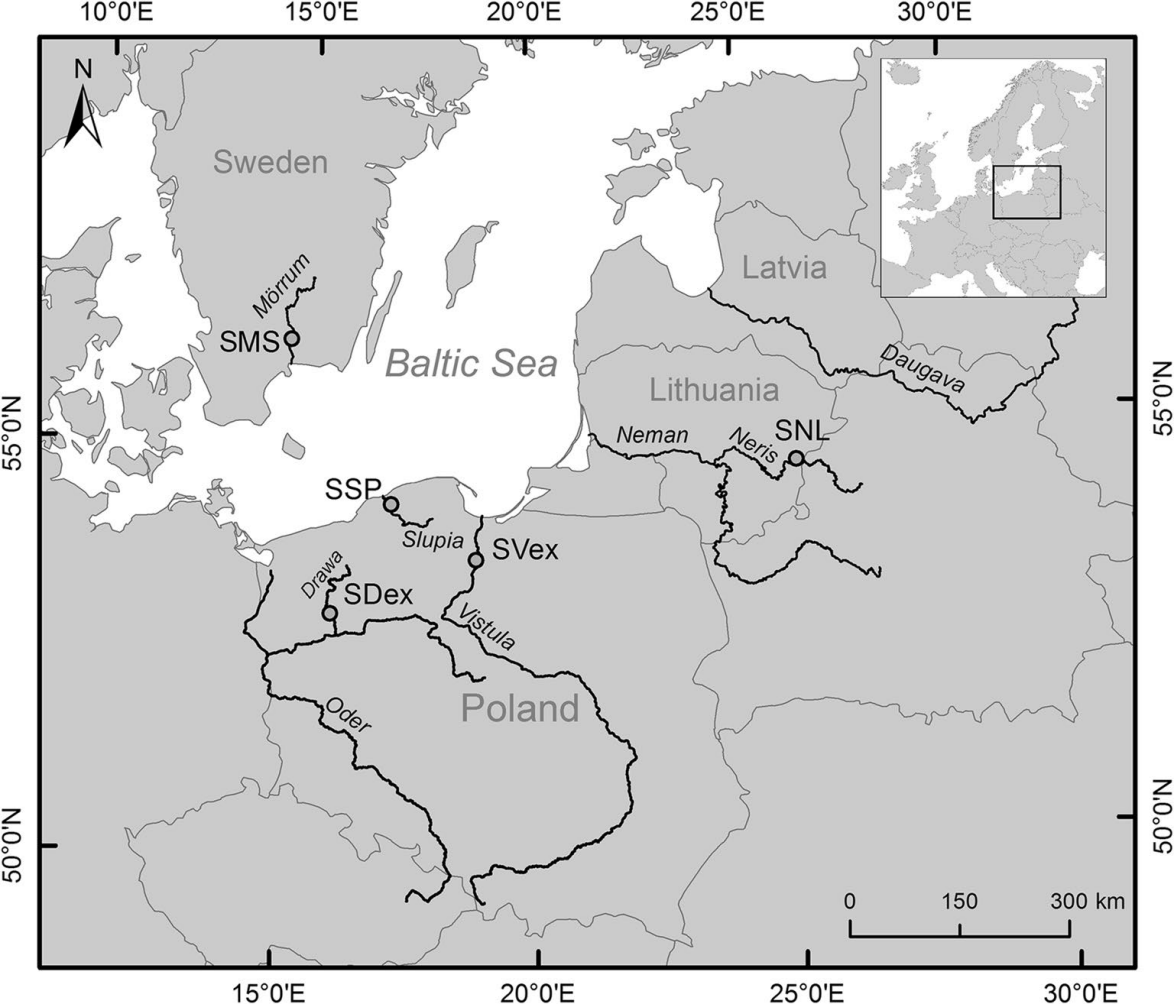
Map of the sites where salmon were sampled in the Southern Baltic area. SSP—Poland, Slupia River; SMS—Sweden, Morrum River; SNL—Lithuania, Neman River; SVex—Poland, Vistula River; SDex—Poland, Oder River

Using the Atlantic salmon Illumina 7K single nucleotide polymorphism (SNP) array as in [11], our aim was to investigate the genetic relationships between Southern Baltic salmon stocks (Oder and Vistula river basins) using preserved archival material and current salmon populations in this region. All methods and sampling are described in detail in Additional file 1 and on Fig. 1.

## Results

Analysis of genotyping data showed that the number of polymorphic SNPs for each population ranged from 874 (Vistula) to 1608 (Morrum) and that the mean number of alleles ranged from 1.571 (Neman) to 1.786 (Morrum). Observed heterozygosity per locus/population ranged from 0.235 (Vistula) to 0.339 (Neman) and expected heterozygosity from 0.241 (Vistula) to 0.32 (Slupia and Neman) (Table 1). Allelic richness A_R_ and private allelic richness P_AR_ ranged from 1.116 to 1.230 and 0.010 to 0.050, respectively. We detected 17 SNPs that deviated from Hardy–Weinberg equilibrium (P ≤ 0.05) after Bonferroni correction for multiple comparisons (Table 1).

**Table 1:** Basic statistics of five salmon populations from the southern Baltic Sea

Population acronyms	NI	NPL	MNA	H_O_	H_E_	A_R_	P_AR_	DHWE*	*F*_IS_
SSP	28	1291	1.63	0.33	0.32	1.20	0.01	1	−0.03
SNL	28	1168	1.57	0.33	0.32	1.18	0.02	0	−0.05
SMS	28	1608	1.78	0.29	0.29	1.23	0.05	0	0
SVex	24	874	1.67	0.23	0.24	1.16	0.04	7	−0.03
SDex	21	904	1.75	0.28	0.29	1.22	0.03	9	−0.03

* After Bonferroni correction (P ≤ 0.05)

*NI* number of individuals, *NPL* number of polymorphic loci, *MNA* mean number of alleles, *H*_*O*_ observed heterozygosity, *H*_*E*_ expected heterozygosity, *A*_*R*_ allelic richness, *P*_*AR*_ private allelic richness, *DHWE* number of loci that deviate from Hardy-Weinberg equilibrium, *F*_IS_ population-specific inbreeding coefficient

SSP = Poland, Slupia River; SMS = Sweden, Morrum River; SNL = Lithuania, Neman River; SVex = Poland, Vistula River; SDex = Poland, Oder River

Population-specific *F*_IS_ estimates were non-significant in all studied populations (P ≤ 0.05), whereas pairwise *F*_ST_ values were significant before and after Bonferroni correction (P ≤ 0.05) for all tests, with higher values between present-day Slupia (SSP) salmon and the extinct Vistula (SVex) salmon (Table 2). By contrast, *F*_ST_ was lowest for the Oder River (SDex) pair. Estimation of global *F*_ST_ by AMOVA for all pairs of loci was equal to 0.2515, which indicates a high level of genetic divergence among the analyzed populations. Overall *F*_IS_ reached −0.0065.

**Table 2:** Genetic diversity indices for the investigated salmon populations

	SSP	SNL	SMS	SVex	SDex
SSP	*408.305*	119.976	118.100	188.257	98.740
SNL	0.236	*370.559*	155.020	125.223	58.974
SMS	0.212	0.269	*471.783*	200.768	113.329
SVex	0.349	0.275	0.343	*282.548*	96.021
SDex	0.200	0.136	0.208	0.227	*377.460*

Below the diagonal are given the *F*_ST_ values for pairwise comparisons of five salmon populations, which were all significant (P = 0.05); on the diagonal in italic characters are given the average numbers of within-population pairwise differences; above the diagonal are given Nei’s genetic distances (*D*_A_)

SSP = Poland, Slupia River; SMS = Sweden, Morrum River; SNL = Lithuania, Neman River; SVex = Poland, Vistula River; SDex = Poland, Oder River

**Fig. 2:**
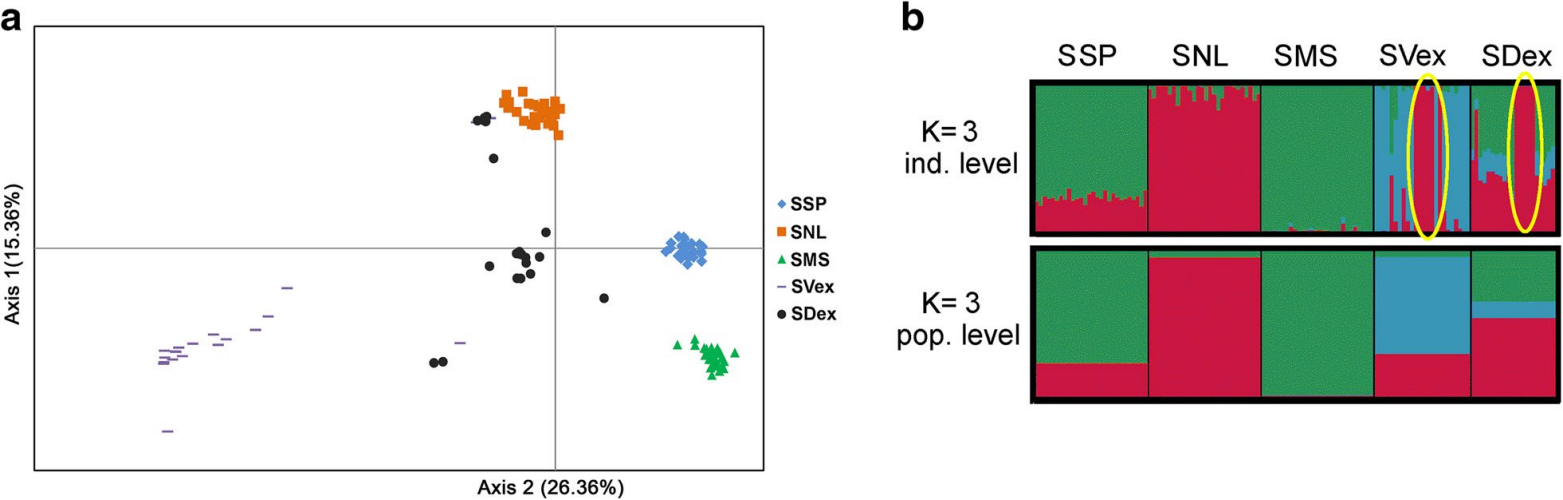
**a** Principal coordinates analysis (PCoA) based on all SNPs. **b** Clustering of 129 specimens from five populations with a putative K value of 3. Each individual is represented by a column divided into K *shades* with each *shade* representing a cluster (*upper bar*, individual level); the *lower bar* represents the results at the population level where each *shade* corresponds to the number of K. *Yellow circles* indicate sub-structuring

The STRUCTURE software was used to examine the relationships among the salmon populations. The maximum value of ∆K was found for K = 3 and the mean log likelihood against K showed a clear plateau at K = 3. Bayesian analysis revealed the existence of substructures in the extirpated populations SDex and SVex (Fig. 2). These sub-clustering phenomena were further investigated by assignment tests, which revealed the presence of potential migrants. Populations from Slupia, Morrum and Neman matched with a 100 % score to their own sample group. Six Vistula specimens from the preserved collection and five specimens from the Drawa River had a genetic makeup that was similar to that found for the current Neman River salmon population; in addition, one SDex fish from the archive collection was genetically related with fish collected in the Morrum River. Results from principal coordinate analysis showed large distances between investigated populations. However, the SNL population was more closely related to SDex than to SVex (Fig. 2).

## Discussion

During the last decade, many new fish passes have been constructed on Pomeranian rivers in the southern Baltic area (e.g. seven in the Slupia River basin) and a previous fish pass on the Włocławek dam in the Vistula River was rebuilt in 2015. These systems allow the passage of Atlantic salmons and have increased their migration but the recovery program that still continues is only moderately successful [11]. In the Baltic Sea area, the percentage of wild stray salmon is relatively low and they only occasionally contribute to spawning nonnative rivers. However due to stocking activity and especially to the release of juveniles in river-mouth areas, the percentage of strayers can increase by up to dozens of percent [12].

Our findings demonstrate that preserved fish scales can be a very valuable source of genomic DNA in population genetic studies. However, analyses based on markers from non-coding regions such as microsatellites often lead to a large amount of allele dropout [13], whereas with SNPs this phenomenon is much less common [14] although archival samples can be affected by a reduction in the number of heterozygous alleles. Johnston et al. [15] reported that genotyping DNA from preserved Atlantic salmon scales was highly successful for samples up to 24 years old. Although the degree of DNA degradation increases with age, we were able to successfully genotype DNA from scales that were older than 50 years. Furthermore, as Johnston et al. [15] indicated, we observed that, with such samples, incorrectly aligned reads occur mainly in the case of multi-site variants, thus analysis of most of this type of genetic variation was not possible.

In our study, the lowest level of heterozygosity, most departures from HWE and the lowest allelic richness were found for the salmon individuals that were sampled from the Vistula River. These results can be explained by a reduction in population size that was inferred from a drop in commercial and research catches prior to complete extinction in the 1960 s [3].

Säisä et al. [9] suggested that the southern Baltic Sea was colonized by salmon from a southern refugium during the Baltic Ice Lake stage since deglaciation occurred very early in the Baltic Sea history. Pre-glacial ice-dammed lakes are also known to have existed in the Neman, Vistula and Oder basins and they may have served as glacial salmon refugia [16, 17]. Our results partially support this theory. Among the rivers studied here, the Oder River was the first deglaciated area about 14.5 ka BP, followed by the Vistula and Neman Rivers about 13.8 to 13.2 ka BP, and finally the Morrum and Daugava rivers between 12.8 and 12.5 ka BP [18]. The Bayesian analysis showed that the populations from the Oder, Vistula and Neman Rivers were separated by relatively large genetic distances, in particular, between those from the Vistula and Oder Rivers, which supports the hypothesis that they originated from geographically close but separated refugia.

Our data showed that the historical salmon population from the Oder River was genetically closer to salmon from the Neman River than from the Vistula River. Salmon from the Vistula River were clearly separated from all other analyzed stocks. Although a mitochondrial DNA study reported that salmon populations from the Neman River clustered with populations from the eastern Baltic Sea including the Daugava River [10], our data did not confirm this result, which may be due to mitochondrial and nuclear markers having different discriminating potential [19].

Results from the STRUCTURE analysis provided evidence of population substructures in the samples from the Vistula and Oder Rivers, which originate from the presence of migrant fish since among the samples from both of these extinct populations, potential migrant fish from the Neman River were detected (PCoA and assignment test). This may be explained by the presence of strayers from the Neman River that could have been caught in Polish rivers or the effect of stocking activities. In our analysis, the salmon population from the Morrum River and the Polish present-day line introduced from the Daugava River stock each formed a cluster, which indicates that the post-glacial origin of the Atlantic salmon from southern Sweden and the origins of the Polish populations from the Vistula and Oder Rivers differ. Since the formation of the southern Baltic rivers occurred much earlier than that of rivers from the Baltic Main Basin, this may explain the differences among Polish stocks and populations from the south Scandinavian Peninsula.

## Conclusions

Native Atlantic salmon populations from the southern Baltic Sea originated from diverse sources after the retreat of the glacial ice sheet. Although the polish historical salmon populations from the Oder and Vistula Rivers clustered together, they were separated by a significant genetic distance. The population from the Oder River was more closely related to the present-day Neman River stock than that from the Vistula River and genetic substructure was revealed in these historical populations. The Polish native populations had a different origin and clustered separately from the Morrum and Daugava River salmon.

## Additional file

10.1186/s12711-016-0208-y Methodology used in this study. This file describes the methods applied in this study [20–29].
