# Supplementary material for: The genetic relationship between extirpated and contemporary Atlantic salmon Salmo salar L. lines from the southern Baltic Sea
Source: Genet Sel Evol. 2016 Apr 1;48:29. doi: 10.1186/s12711-016-0208-y (PMC4818505; doi:10.1186/s12711-016-0208-y)
Supplement: Supplementary file 1 — 10.1186/s12711-016-0208-y Methodology used in this study. This file describes the methods applied in this study [20–29]. [file 12711_2016_208_MOESM1_ESM.docx]

**Additional file 1**

**Materials and methods**

This file provides information about the methods with references [20-29].

**Sampling and DNA extraction**

Key to samples abbreviation: SSP – Salmon, Słupia River, Poland; SNL – Salmon, Neman River, Lithuania; SMS – Salmon, Morrum River, Sweden; SVex – Salmon, Vistula River, extinct, SDex – Salmon, Drawa River, extinct.

Samples from extinct Polish populations from the Vistula and Oder rivers (Fig. 1) were extracted from archival scales that had been previously stored for age reading. All samples from native Vistula salmon came from specimens that had been caught directly in the river about 50 to 70 km upstream from Gdansk Bay between 1951 and 1957. Archival scales representing original Oder River salmon were sampled from progeny of specimens found in the Drawa River, a tributary of the Oder and released during tagging experiments between 1965 and 1971.

Archival samples were treated individually, depending on tissue source (mainly scales). However, where possible, blood and mucus from storage envelopes were washed out and added to the incubation together with the scales. Sherlock AX kit (A&A Biotechnology, Gdynia, Poland) according to modified manufacturer’s guidelines were used for archival samples. Samples were then analyzed in 1.0 % agarose gels and quantified with a NanoDrop spectrophotometer. The quality of genomic DNA from historical samples was also checked by PCR reaction with two microsatellite loci Ssa85 and Ssa197. Samples were then genotyped using an Atlantic salmon Illumina SNP-chip [20] at the Centre for Integrative Genetics (CIGENE), Norway. In addition, genotyping results of samples of present-day populations of salmon published by Poćwierz-Kotus et al. [11] were used for population genetic analyses in this study. In their study, samples were taken from 3 locations in the southern Baltic: wild parr from the Slupia River, Poland, wild smolts from River Morrum, Sweden and wild adults from the Lithuanian River Neris – a tributary of the Neman River.

**SNP genotyping and validation**

Among 5568 genotyped SNPs, 3041 were successfully amplified both for contemporary and archival samples. Unsuccessfully genotyped loci in historical DNA mainly belonged to multi-site variants such as MSV3, MSV4 or MSV5 and this type of loci was discarded. Paralogous site variant (PSV), monomorphic, null alleles and mitochondrial SNPs were also excluded from the analysis. An accepted threshold of missing data rate was assessed at 8 5%. On the basis of analysis of allele frequency, 996 SNPs with a MAF (minor allele frequency) with an adopted threshold equal to 0.01 were identified and they were excluded from further analysis. The set of polymorphic SNPs was counted at 2045 loci.

**Statistical analysis**

Departures from Hardy–Weinberg equilibrium were tested using the Markov chain Monte Carlo random algorithm in Arlequin 3.1. Also, population-specific *F*_IS_, pairwise weighted *F*_ST_ values over all loci based on the number of different alleles were identified using this software. A sequential Bonferroni type method was employed to correct for multiple significance tests. Overall, F-statistic was estimated by analysis of molecular variance (AMOVA) implemented in Arlequin 3.1 [21]. HP-RARE was used to calculate allelic richness and richness of private alleles [22].

STRUCTURE 2.3.3 was used to detect genetic structure and gene flow [23]. The Evanno method (∆K) was used [24] to infer the true number of clusters (K), based on the rate of change in log probability between consecutive K values, which ranged from K = 1 to 6 with a burn-in and MCMC length of 10,000 each for five runs per K-value (100,000 burn-in and 200,000 MCMC replicates when analyzing for hierarchical within-cluster structure). STRUCTURE HARVESTER program was used for K value testing [25]. The CLUMPP 1.1.2 [26] software package was used to find the optimal alignment of the results from the five replicate cluster analyses. Full search algorithm and 10,000 random permutations were applied. Genetic structure was also analyzed by performing principal coordinate analysis implemented in GenAlEx 6.502 software [27].

Tests of assignment to determine the most likely origin for all 129 salmon individuals were conducted using GeneClass2 [28] with an allele frequency-based method [29].
